# Contrast-enhanced ultrasound (CEUS) in characterization of inconclusive cervical lymph nodes: a meta-analysis and systematic review

**DOI:** 10.1038/s41598-022-11542-9

**Published:** 2022-05-12

**Authors:** Paul Spiesecke, Konrad Neumann, Katharina Wakonig, Markus H. Lerchbaumer

**Affiliations:** 1 Department of Radiology, Interdisciplinary Ultrasound Center, Charité – Universitätsmedizin Berlin, corporate member of Freie Universität Berlin, Humboldt-Universität Zu Berlin, and Berlin Institute of Health, Charité -Universitätsmedizin Berlin Campus Charité Mitte, Charitéplatz 1, 10117 Berlin, Germany; 2 Institute of Biometry and Clinical Epidemiology, Charité – Universitätsmedizin Berlin, corporate member of Freie Universität Berlin, Humboldt-Universität Zu Berlin, and Berlin Institute of Health, Berlin, Germany; 3 Department of Otorhinolaryngology, Charité – Universitätsmedizin Berlin, corporate member of Freie Universität Berlin, Humboldt-Universität Zu Berlin, and Berlin Institute of Health, Berlin, Germany

**Keywords:** Cancer imaging, Metastasis, Ultrasonography

## Abstract

Lymph node metastases are common in malignant neoplasms of head and neck. Since cervical lymph nodes (cLN) are localized superficially, ultrasound (US) represents the primary imaging modality. The aim of the study is to report the value of US and contrast-enhanced ultrasound (CEUS) and their diagnostic confidence in the characterization of inconclusive cLN. A systematic review was performed using the literature data base PubMed. Results were filtered (published in a peer-reviewed journal, full-text available, published within the last ten years, species human, English or German full-text) and inclusion criteria were clearly defined (cohort with lymphadenopathy or malignancy in head and neck ≥ 50 patients, histological confirmation of malignant imaging findings, performance of CEUS as outcome variable). The results were quantified in a meta-analysis using a random-effects model. Overall, five studies were included in qualitative and quantitative analysis. The combination of non-enhanced US and CEUS enlarges the diagnostic confidence in the characterization of lymph nodes of unclear dignity. The pooled values for sensitivity and specificity in the characterization of a malignant cervical lymph node using US are 76% (95%-CI 66–83%, I^2^ = 63%, *p* < 0.01) and 80% (95%-CI 45–95%, I^2^ = 92%, *p* < 0.01), compared to 92% (95%-CI 89–95%, I^2^ = 0%, *p *= 0.65) and 91% (95%-CI 87–94%, I^2^ = 0%, *p* = 0.40) for the combination of US and CEUS, respectively. Consistent results of the included studies show improved diagnostic performance by additional CEUS. Nevertheless, more prospective studies are needed to implement CEUS in the diagnostic pathway of cLN.

## Introduction

Cervical lymph node (cLN) metastases are common in patients with malignant head and neck neoplasms especially in squamous cell carcinomas (HNSCC) of the upper aerodigestive tract^1^. In only 1% of the cases, the primary cause of cLN metastasis is found extracervical, among them in tumors of breast, lung, gastrointestinal tract, urogenital tract and central nervous system^1^. Besides cLN metastases and benign causes like infections, malignant lymphoma can be a cause of cervical lymphadenopathy as well and must be taken into consideration at differential diagnosis^1,2^. In 2016, neoplasia of oral cavity and pharynx were the eighth most frequent cancer in men and thyroid neoplasia was the fifth common cancer in women, respectively in the United States^3^.

Criteria for malignant cLN transformation in B-mode US are round shape, calcifications and heterogenic with hyperechogenic or cystic changes^4^. Leng et al. reported a sensitivity of 84% (95% CI: 67–93%) and specificity of 93% (95% CI: 90–95%) using US investigating the detection of cLN metastases in patients with esophageal carcinoma using a cut-off value of 5 mm cLN size^5^.

Over the last decade, the use of contrast-enhanced ultrasound (CEUS) for characterization of focal lesions especially in liver and kidney increased, representing an additional contrast-enhanced modality beside CT (computed tomography) and MRI (magnetic resonance imaging) in the diagnostic pathway for detection of malignant lesions^6^.

The present article highlights the additional diagnostic value of CEUS in inconclusive cLN by a systematic review of the current literature with meta-analysis.

## Material and methods

### Data source and literature search

The systematic literature research was performed by using the literature data base PubMed. Allowing a preferably extensive search, the request was composed with synonyms (Table 1).

**Table 1:** Scheme presenting the search request of the systematic review.

Entity	AND		
OR	CEUS	Lymph node	Cervical
	Contrast-enhanced ultrasound	Lymph nodes	Neck

Hence resulting in the following search request:

*((CEUS) OR (contrast-enhanced ultrasound)) AND ((lymph node) OR (lymph nodes)) AND ((cervical) OR (neck))*

The results were restricted using additional filters in PubMed: Articles being available in full text (until the 17th March 2020), published maximum 10 years ago (reflecting the rapid technical progress in CEUS), examining humans and written in English or German. All results were assessed by two authors (*blinded*) independently.

### Selection of studies and criteria

Only original scientific articles being published in peer-review journals were considered. A further inclusion criterium was the patient cohort which must consist of patients with (i) cervical lymphadenopathy, (ii) malignancy in head and neck and (iii) must include a minimum of 50 patients. Histological confirmation was taken as gold standard acquired by biopsy or surgical removal. In case of benign initial imaging result, an additional sonographic follow-up of at least two years was permitted due to an overall low number of available studies addressing the issue of the present review. The studies can be designed both pro- and retrospectively. The target variable must be the diagnostic performance of CEUS, respectively in comparison to prior US.

### Assessment of the risk of bias

The risk of bias was assessed using the QUADAS-2 (Quality Assessment of Diagnostic Accuracy Studies)^7^. Therefore, the included studies were evaluated concerning patient selection, the index test, the reference standard as well as flow and timing^7^.

### Review of single qualitative and quantitative parameters

Individual parameters used the differentiation of malignant and benign cLN in US, CEUS and connected post-processing analyses (such as time-intensity-curve [TIC] models) were extracted from the included studies if parameters showed a significance level of α ≤ 0.05. The extraction of diagnostic accuracy from the original publications (four-field tables) was either performed by number of cLN or patients included, depending on the authors individual choices.

### Meta-analysis

With the aim of quantifying the results obtained (sensitivity and specificity of the modalities to be compared), a meta-analysis was carried out comparing the modalities US, CEUS and combination of US and CEUS. For this purpose, the function “metaprop” from the R package “meta” was used and modelling was performed using a random intercept logistic regression model (random effects model)^8^. I^2^ was determined as a measure of heterogeneity as well as τ^2^ as a measure of between-study variance with p-value. The calculated parameters were interpreted using accepted references^9,10^.

## Results

### Choice of the included studies

**Figure 1:**
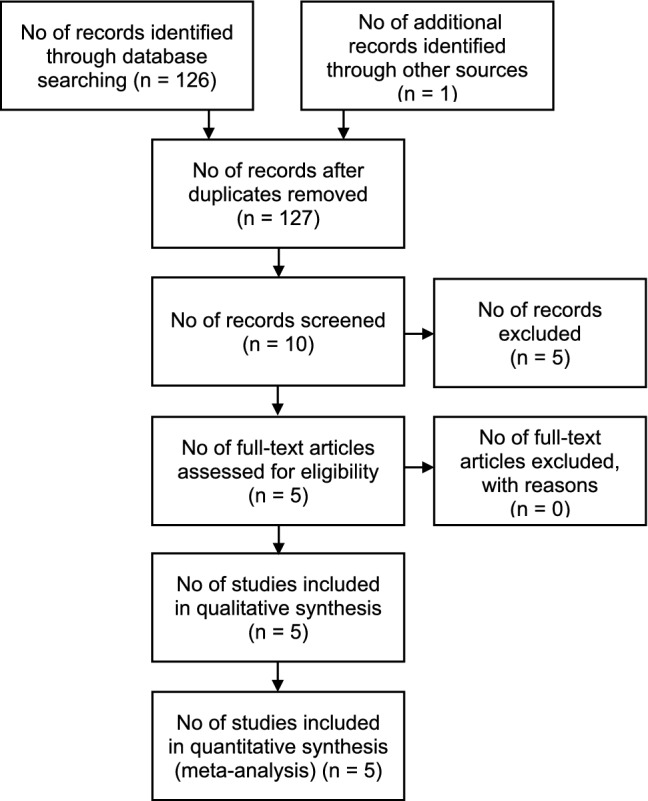
PRISMA-scheme of the systematic literature review. Presented is the PRISMA-scheme of the systematic literature review, inspired by Moher et al.^11^.

In the query a total of n = 246 publications were obtained. After application of the described filters in PubMed, n = 126 results remained. The selection procedure is visualized in a flow-chart which is based on the PRISMA-scheme (Preferred Reporting Items for Systematic Reviews and Meta-Analyses) (Fig. 1)^11^.

Beside the 126 results found via PubMed, a publication of Chen et al. has been taken into consideration due to author’s precognition^12^. From these overall 127 results, ten articles have been preselected due to thematic accordance via screening of the abstracts^12–21^.

After screening, five studies were excluded regarding the mentioned inclusion criteria. Mansour et al. investigated ultrasonographic hypoechoic head and neck lesions with less than 50 patients with cLN disease included^16^. Zhan et al. did not examine the diagnostic performance of CEUS, but performed a multivariate analysis predicting LN metastasis by examining papillary thyroid cancer with CEUS^13^. Similar, Zhang et al. explored the prediction of LN metastases by examining papillary thyroid cancer with CEUS and must be excluded since its target variable is not the diagnostic performance of CEUS in the characterization of cLN^15^. Dudau et al. published a study examining if CEUS can distinguish malignant from reactive LN in patients with head and neck cancer, but had to be excluded due to the small number of patients included (n = 17)^20^. Wendl et al. described preoperative use of CEUS and MRI in characterizing LN, but had to be excluded due to a small cohort of only 10 patients as well^21^.

Finally, five remaining articles were assessed for their eligibility. All of them were judged as permissible for qualitative and quantitative assessment fulfilling all inclusion criteria mentioned above^12,14,17–19^.

### Results of studies included

**Figure 2:**
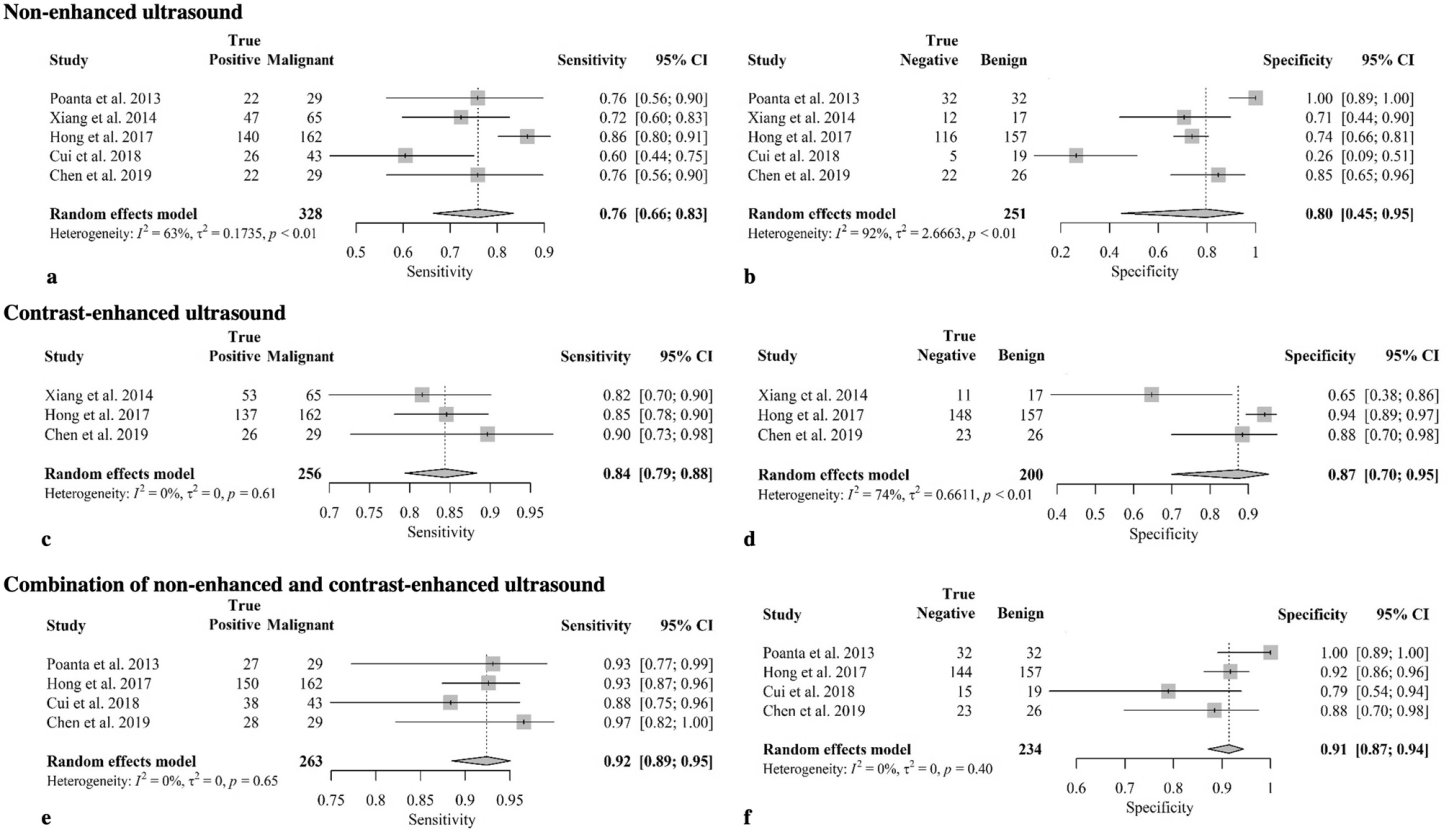
Summary of the diagnostic accuracy of the compared modalities and associated forest plots. The forest plots of the individual compared modalities belonging to the meta-analysis are shown. A distinction is made between non-enhanced US (**a**, **b**), CEUS only (**c**, **d**) and their combination (**e**, **f**). In each group, the achieved sensitivities (**a**, **c**, **e**) and the specificities (**b**, **d**, **f**) are shown. The width of the individual plots lines is determined by the 95%-CI.

An overview of the included studies outlining their characteristic is given in Table 2. All studies used 1.0–2–4 mL of SonoVue® (Bracco, Milan, Italy) as contrast agent for CEUS. Quantitative results are presented in Fig. 2 and qualitative results are presented in Table 4.

**Table 2:** Overview and synopsis of the included studies.

References	Population	Confirmation	Cohort size	Result	Study design	Possible bias
Poanta 2014^19^	Patients with palpable mass in cervical region	Histology	61 patients, 1 cLN each (29 malignant, 32 benign)	The combination of gray scale US, Doppler and CEUS enlarges the diagnostic performance	Prospective	Calculation of sensitivity and specificity with ROC analysis, not by blinded reading of the pictures
Xiang 2014^18^	Patients with thyroid cancer who underwent lateral neck dissection	Histology	82 patients, in total 102 cLN (71 malignant, 31 benign)	CEUS is superior to US according to diagnostic accuracy	Retrospective	There was no statistical significance for the non-enhanced US findings shown, but a comparison of the diagnostic performance of CEUS and US
Hong 2017^14^	Patients with thyroid nodules (Bethesda-system ≥ category IV) or with thyroid cancer in history, only metastases from papillary thyroid cancer were included	Histology in malignant cLN; in benign cLN, a ≥ 2 years after thyroidectomy US follow-up was used as confirmation besides histology	253 patients, in total 319 cLN (162 malignant from PTC, 157 benign)	Combination of US and CEUS enlarges the diagnostic accuracy	Prospective	Part of the exclusion criteria was another metastasis than one of a PTC, but this review is not limited to cLN with PTC-metastasis; In benign cLN also US follow-up was used as confirmation, whereas histology is the gold standard
Cui 2018^17^	Patients with cervical lymphadenopathy who have been diagnosed with tuberculosis or malignancy	Histology or cytology (in case of tuberculosis)	62 patients, 1 cLN each (43 malignant, 19 benign tuberculous)	CEUS and TIC enlarge the diagnostic accuracy towards native US only	Not given	Comparison of tuberculous and malignant LN after confirmation; not all patients received the same reference standard
Chen 2019^12^	Analysis of patients with histological confirmation of a reactive/normal or a malignant cLN with PTC-metastasis, US examination not later than one month before probe extraction	Histology	46 patients, in total 55 LN (29 malignant, 26 benign)	Combination of US and CEUS enlarges the diagnostic accuracy; Quantitative CEUS-parameters were statistically not significant	Retrospective	Part of the exclusion criteria was another metastasis than one of a PTC, but this review is not limited to cLN with PTC-metastasis

Poanta et al. investigated the role of CEUS in the differentiation between benign and malignant cLN in the differential diagnosis of superficial lymphadenopathy. The authors used receiver operating characteristic (ROC) analysis to receive sensitivity and specificity of sonographic parameters. The authors found CEUS to be especially useful in case of uncertain B-Mode-US investigation results^19^.

On the other hand, Xiang et al. researched the diagnostic value of CEUS in patients with thyroid cancer. There was no statistical analysis according to statistical significance of qualitative non-enhanced sonographic findings except cLN diameter performed, so only CEUS’ enhancement pattern is presented in this review. The authors sum up centripetal enhancement pattern, microcalcifications as well as perfusion defects as specific malignant criteria^18^.

Hong et al. also studied cLN in patients with papillary thyroid cancer (PTC) metastases. The results are in line with the previous studies, as centripetal enhancement, hyperenhancement and perfusion defects were also found as significant features. In addition, the authors emphasized the importance of a ring-enhancing margin in malignant cLN^14^.

Cui et al. examined the diagnostic value of CEUS in the differentiation between metastatic and tuberculous cLN. As described by Poanta et al. quantitative TIC parameters were also found to be significant in this particular cohort. Diagnostic accuracy was particularly enhanced by the use of CEUS, which highlights the diagnostic difficulties associated with tuberculous cLN^17^.

Chen et al. like Hong et al. studied a cohort with cLN metastasis from PTC. Regarding the qualitative CEUS characteristics, the results overlap with previous studies, but the authors did not find any quantitative CEUS parameters to be statistically significant^12^.

### Assessment of the risk of bias

The assessment regarding the risk of bias in the included studies results in an overall picture of applicability. An overview of the categories and studies following the suggestions of QUADAS-2^7^ is presented in Table 3.

**Table 3:** Assessment of the risk of bias of the included studies.

References	Risk of bias				Applicability concerns		
	Patient selection	Index test	Reference standard	Flow and timing	Patient selection	Index test	Reference standard
Poanta et al. 2014	Low	Low	Low	Low	Low	Low	Low
Xiang et al. 2014	Low	Low	Low	Unclear	Low	Low	Low
Hong et al. 2017	Low	Low	Unclear	High	Low	Low	Low
Cui et al. 2018	High	Low	Low	Low	High	Low	Low
Chen et al. 2019	Low	Low	Low	Low	Low	Low	Low

Presented are the assessed risks of bias and concerns regarding applicability of the studies following QUADAS-2^7^ procedure. The indications obtain the degree of risk and the concerns of applicability, respectively.

The studies by Poanta et al. and Chen et al. were assessed to fit the question of the review well showing low risk of bias in all assessed categories^12,19^. In the study by Xiang et al. the diagnostic accuracy was evaluated according to the number of patients included, but not according to the larger number of cLN examined—nevertheless, this study fits well with the research question of the review^18^. In the study by Hong et al. it is striking that not all patients received the same reference standard (although it was not stated how many patients were allocated to the benign cohort solely by follow-up)^14^. However, it is estimated that an imaging follow-up over two years for initial suspicion of a benign cLN represents a pragmatic approach that overall fits well with the question of the review^14^. The study by Cui et al. generally fits well with the question of the review, but it examines a pre-selected cohort in which only tuberculous cLN were included in the benign cohort^17^. As a result, the patient selection is at risk of bias. Nevertheless, the present review is not limited to tuberculous cLN in the case of benign cLN—which, however, represents a decisive component of this group^17^.

Despite the partial risk of bias, all studies are included in the qualitative and quantitative analysis, due to the limited number of available studies and also because Whiting et al.^7^ describe this as preferable. Possible heterogeneities are discussed separately.

### Qualitative results of the studies

The qualitative results of the studies—in the sense of image-morphological criteria of malignant cLN—are presented In Table 4. Therefore, statistically significant (*p* ≤ 0.05) characteristics of metastatic cLN are listed.

**Table 4:** Statistically significant features for malignant cervical lymph nodes found in the included studies.

Study	Non-enhanced US	CEUS/TIC
Poanta 2014^19^	L/S-ratio ≤ 2 Hypoechoic Hilum hard to see/abscent Inhomogeneous internal structure Irregular/blurred margins Peripheral/mixed vascular pattern Chaotic vascular pattern Multiple pedicullus	Inhomogeneous contrast enhancement/no enhancement Reduced derived peak intensity Enlarged area under the curve Reduced regional blood volume
Xiang 2014^18^		Heterogenous enhancement Perfusion defects Microcalcification Centripetal or hybrid enhancement
Hong 2017^14^	L/S-ratio < 2 Ill-defined margin (poorly circumscribed, blurred, irregular or with angular margins) Absent hilum Hyper-echogenicity Heterogeneity Cystic necrosis Calcification Mixed or peripheral vascularity	Centripetal or asynchronous perfusion Nonor hyperenhancement Heterogenous enhancement Perfusion defect Ring-enhancing margin
Cui 2018^17^	No significant differences between malignant and tuberculous LN found	Centripetal enhancement No apparent notch in TIC Shallow descending curve in TIC Quantitative TIC-parameters
Chen 2019^12^	L/S-ratio < 2 Poorly defined margin Hyperechoic Absent hilum Calcification	Peripheral or mixed blood flow distribution Centripetal perfusion Peripheral or mixed (varied degrees of enhancement, including nonenhancement, mixed in different parts of the LN) enhancement Enlarged range on CEUS compared to US

CEUS denotes contrast-enhanced ultrasound, L/S-ratio denotes the ratio of long- and short-axis diameter, TIC denotes time intensity curve, US denotes ultrasound.

### Meta-analysis

In addition to the qualitative results of the individual included studies as presented in Table 4, the pooled data calculated with a random effects model for the three different imaging modalities investigated are shown in Fig. 2.

For non-enhanced US, the pooled sensitivity was 76% (95%-CI 66–83%, I^2^ = 63%, *p* < 0.01) and the specificity 80% (95%-CI 45–95%, I^2^ = 92%, *p* < 0.01) showing a high heterogeneity.

Considering CEUS alone in the determination of the dignity of cLN, three of the five included studies provided results that showed increased pooled diagnostic performance. The sensitivity reached a value of 84% (95%-CI 79–88%, I^2^ = 0%, *p* = 0.61) and the specificity 87% (95%-CI 70–95%, I^2^ = 74%, *p* < 0.01). Heterogeneity decreased sharply in the case of sensitivity, whereas it remained high in the case of specificity.

A total of four of the included studies investigated diagnostic accuracy using the combination of US and CEUS. This resulted in the highest diagnostic accuracy of the present analysis represented by a sensitivity of 92% (95%-CI 89–95%, I^2^ = 0%, *p* = 0.65) and specificity of 91% (95%-CI 87–94%, I^2^ = 0%, *p* = 0.40). Overall, the heterogeneity can be assessed as low.

## Discussion

The results of the present review can be summarized as follows: (I) The diagnostic performance of cLN sonography rises by using CEUS instead of US, whereas its combination constitutes the best diagnostic option among the modalities examined in this meta-analysis and is accompanied by the lowest heterogeneity among the studies included. (II) One included study found no statistically significant characteristics in non-enhanced US to determine a cLN entity in subgroup of metastatic versus tuberculous lymph nodes^17^. Three other studies included found different and partly contradicting results, whereas in particular a L/S-ratio < 2 (ratio of long-axis and short-axis diameter) and atypical margins form the overlapping set^12,14,19^. (III) As a qualitative CEUS characteristic, all but one study found inhomogeneous enhancement as a statistically significant pattern^12,14,18,19^. The studies which included quantitative CEUS characteristics produced different results in terms of statistical significance of TIC parameters^12,17,19^.

In 2014, Ying et al. published a systematic literature review examining the value of US, CEUS and elastography in the assessment of cLN^22^. They state that the improvement of the diagnostic performance by adding CEUS to US is evaluated as controversial with inconsistent results of the included studies^22^. Our results show no contradiction with enlarging sensitivity and specificity in all included studies also presented for the pooled values in the meta-analysis. Mei et al. investigated in 2018 in a meta-analysis the diagnostic accuracy of CEUS in differential diagnosis of superficial LN including head and neck area as well as axillar and groin LN^23^. In conclusion, they found a sensitivity of 88% (95%-CI 83–92%) and a specificity of 80% (95%-CI 74–85%) for CEUS which is a lower accuracy compared to the present results^23^. Additionally, we performed a comparison of US, CEUS and their combination. Nevertheless, the external validity regarding CEUS’ diagnostic accuracy in differential diagnosis of cLN can be assessed as sufficient.

In particular, analysis of perfusion using TIC parameters offers the opportunity to quantify sonographic findings, which could decrease one of the well-known disadvantages of diagnostic US—the operator dependency—by generating objective parameters. In this context, however, the partly contradictory results regarding dedicated TIC parameters of the included studies of the present review must be considered^12,17,19^. A possible reason for these results can be the partially different concept of data collecting: Cui et al. compared the cLN’s perfusion with the perfusion of the carotid artery’s perfusion, whereas Chen et al. captured parameters which are independent of the surrounding tissue’s perfusion such as peak intensity, time to peak, mean transit time and rise time^12,17^. Poanta et al. also used parameters that were independent of the enhancement of the environment^19^. So, these parameters could balance the examiner dependance in future, but for this purpose, it is necessary that future studies collect corresponding parameters as uniformly as possible.

Even if there is greater evidence for the usability of TIC parameters in the future, their sole use should be questioned. Ultimately, these parameters—even if their objectivity seems tempting—should always be correlated with US characteristics and patients’ history or paraclinical findings. On the one hand, CEUS is always preceded by a US examination, and on the other hand, our results suggest that the combination of both modalities should generally be considered. At the same time, we showed a significantly reduced heterogeneity of the included studies for the combined use of US and CEUS, which increases the significance of this effect (Fig. 2).

However, it has to be mentioned that the heterogeneity of the studies in the field of B-Mode US is mainly due to the deviating sensitivity and specificity reported by Cui et al.^17^ The increase in accuracy in combination with CEUS is then striking, so that the study joins the results of the other studies with a heterogeneity of I^2^ = 0% for sensitivity and specificity, respectively.

Of course, differentiation of tuberculous and malignant LN is particularly difficult. Moon et al. emphasize the particular importance of a correct diagnosis of tuberculous lymphadenitis, as this requires special therapy^24^. The authors investigated a specific US study protocol evaluated in an endemic area showing a sensitivity and specificity > 90% for the diagnosis of nontuberculous benign, tuberculous and malignant LN, respectively^24^. This is another indication of the advantageousness of using standardized protocols, as discussed above for TIC.

A general benefit of CEUS among tomographic contrast-enhanced imaging modalities is the low adverse-event rate and pulmonary elimination of injected microbubbles: Tang et al. describe in retrospective study containing more than 30 000 patients CEUS’ contrast agent as good and quantify the rate of side effects to 0.020%^25^. Piscaglia et al. come to a similar result and state the rate of side effects after evaluation of more than 23 000 patients to 0.0086%^26^.

Additionally, contrast agent of CEUS can be given to patients with chronic renal insufficiency as well since it is eliminated by the lungs^27^. Concerning iodine-containing contrast agents of CT, the incidence of contrast-induced nephropathy (CIN) has been described to be 2–12%^28^. Moreover, these contrast agents cause complicated application in patients with hyperthyroidism—additionally, in clinical practice, in younger patients the application of CT scans is more restrained because of the associated radiation exposure.

Even though these problems are completely avoided with MRI, there is a more complicated handling of imaging in patients with claustrophobia—whose event rate was quantified at 9.8%^29^—or in patients with pacemaker. This underlines once again why, given the good pooled diagnostic accuracy of the present review, the examination of cLN by CEUS should be pursued, as this modality can facilitate and expedite procedures. As recently published, CEUS showed superior cost effectiveness compared to contrast-enhanced CT and MRI in the characterization of focal lesions on the example of cystic renal lesions^30^.

Despite the promising results and the low calculated heterogeneity in the case of the combination of US and CEUS, the present review is primarily limited regarding the low number of included studies and therefore the low number of patients examined. However, this limitation can be somewhat appeased: Jackson et al. described that at least five studies are needed to map into a random effects model with an overall larger effect than the individual included studies^31^. Nevertheless, each of the included studies also described an increase in diagnostic accuracy using CEUS (Fig. 2).

Uniting the small patient numbers and the promising results, larger prospective studies are needed to confirm present results. Newer applications such as shear wave elastography (SWE) should also be captured to generate more quantitative sonographic parameters. Since this tool is easy to perform and free of side effects, it should be registered in an approach of multiparametric ultrasound (mpUS) as well^6^.

Indeed, mpUS would not remove histology as gold standard, but could reduce the number of necessary invasive diagnostic interventions.

In conclusion, the present review shows an increased diagnostic accuracy when adding CEUS to standard US examinations in the assessment of inconclusive cLN. If further prospective studies show congruence, they could form the foundation for an improved sonographic characterization.

## Abbreviations

CEUS: Contrast-enhanced ultrasound
cLN: Cervical lymph node
CT: Computed tomography
HNSCC: Head and neck squamous cell carcinoma
LN: Lymph node
MRI: Magnetic resonance imaging
PRISMA: Preferred reporting items for systematic reviews and meta-analyses
PTC: Papillary thyroid cancer
QUADAS: Quality assessment of diagnostic accuracy studies
ROC: Receiver operating characteristic
SWE: Shear wave elastography
TIC: Time intensity curve
US: Ultrasound
